# Sedation and analgesia in post-cardiac arrest care: a post hoc analysis of the TTM2 trial

**DOI:** 10.1186/s13054-025-05461-0

**Published:** 2025-06-17

**Authors:** Ameldina Ceric, Josef Dankiewicz, Tobias Cronberg, Joachim Düring, Marion Moseby-Knappe, Martin Annborn, Teresa L. May, Matthew Thomas, Anders Morten Grejs, Christian Rylander, Jan Belohlavek, Pedro Wendel-Garcia, Matthias Haenggi, Claudia Schrag, Matthias P. Hilty, Thomas R. Keeble, Matt P. Wise, Paul Young, Fabio Silvio Taccone, Chiara Robba, Alain Cariou, Glenn Eastwood, Manoj Saxena, Susann Ullén, Gisela Lilja, Janus C. Jakobsen, Anna Lybeck, Niklas Nielsen

**Affiliations:** 1 Anesthesia & Intensive Care, Department of Clinical Sciences, Lund University, Skane University Hospital, Carl Bertil Laurels Gata 9, 214 28 Malmö, Sweden; 2 Cardiology Department, Lund University, Skåne University Hospital Lund, Lund, Sweden; 3 Neurology, Department of Clinical Sciences Lund, Lund University, Skåne University Hospital, Lund, Sweden; 4 Department of Clinical Sciences, Anaesthesia and Intensive Care, Lund University, Skåne University Hospital, Malmö, Sweden; 5 Department of Clinical Sciences Lund, Neurology and Rehabilitation, Lund University, Lund, Sweden; 6 Department of Clinical Sciences, Anaesthesia and Intensive Care, Lund University, Helsingborg Hospital, Helsingborg, Sweden; 7 Department of Critical Care, Maine Medical Center, Portland, ME USA; 8 Department of Intensive Care, University Hospitals Bristol and Weston, Bristol, UK; 9 Department of Intensive Care Medicine, Aarhus University Hospital, Aarhus N, Denmark; 10 Department of Clinical Medicine, Aarhus University, Aarhus N, Denmark; 11 Anaesthesiology and Intensive Care, Department of Surgical Sciences, Uppsala University and Uppsala University Hospital, Uppsala, Sweden; 12 2Nd Department of Internal Mediciny, Cardiovascular Medicine, General University Hospital, and 1St Medical School in Prague, Prague, Czech Republic; 13 Institute of Heart Diseases, Wroclaw Medical University, Wroclaw, Poland; 14 Institute of Intensive Care Medicine, University Hospital Zurich, Rämistrasse 100, 8091 Zurich, Switzerland; 15 Department of Intensive Care Medicine, University Hospital Bern, University of Bern, Bern, Switzerland; 16 Intensive Care Department, Kantonsspital St. Gallen, St. Gallen, Switzerland; 17 Anglia Ruskin School of Medicine, Essex Cardiothoracic Centre, MSE NHS Trust, Essex, UK. MTRC, Chelmsford, Essex, UK; 18 Adult Critical Care, University Hospital of Wales, Cardiff, UK; 19 Intensive Care Unit, Wellington Hospital, Wellington, New Zealand and Medical Research Institute of New Zealand, Wellington, New Zealand; 20 Department of Intensive Care, Hôpital Universitaire de Bruxelles, Université Libre de Bruxelles, Brussels, Belgium; 21 IRCCS Policlinico San Martino; Genoa, Italy and Dipartimento Di Scienze Chirurgiche Diagnostiche E Integrate, University of Genoa, Genoa, Italy; 22 Medical ICU Cochin University Hospital, AP-HP Centre Université Paris Cité, Paris, France; 23 The Australian and New Zealand Intensive Care Research Centre, Monash University, Melbourne, Australia; 24 Division of Critical Care and Trauma, George Institute for Global Health, and Bankstown-Lidcombe Hospital, South Western Sydney Local Health District, Sydney, Australia; 25 Skåne University Hospital Lund, Lund University and Clinical Studies Sweden - Forum South, Skåne University Hospital, Lund, Sweden; 26 Neurology Department of Clinical Sciences, Lund University, Lund, Sweden; 27 Copenhagen Trial Unit – Centre for Clinical Intervention Research, Rigshospitalet, Copenhagen University Hospital, Copenhagen, Denmark; 28 The Faculty of Health Sciences, Department of Regional Health Research, University of Southern Denmark, Odense M, Denmark; 29 Department of Clinical Sciences, Anaesthesia and Intensive Care, Skåne University Hospital, Lund University, Lund, Sweden

**Keywords:** Cardiac arrest, Targeted temperature management, Sedation, Seizures, Propofol, Midazolam

## Abstract

**Background:**

The routine use of sedation and analgesia during post-cardiac arrest care and its association with clinical outcomes remain unclear. This study aimed to describe the use of sedatives and analgesics in post-cardiac arrest care, and evaluate associations with good functional outcome, survival, clinical seizures, and late awakening.

**Methods:**

This was a post hoc analysis of the TTM2-trial, which randomized 1900 out-of-hospital cardiac arrest patients to either normothermia or hypothermia. In both groups, deep sedation (Richmond Agitation and Sedation Scale ≤ -4) was mandatory during the 40-h intervention. Cumulative doses of sedatives and analgesic drugs were recorded within the first 72 h from randomization. Outcomes were functional outcome (modified Rankin Scale) and survival status at 6 months, occurrence of clinical seizures during the intensive care stay, and late awakening (Full outline of unresponsiveness motor score of four 96 h after randomization). Cumulative propofol doses were divided into quartiles (Q1-Q4). Logistic regression models were used to assess associations between sedative doses and functional outcome and survival, clinical seizures, and late awakening, adjusting for the severity of illness and other clinical factors influencing sedation.

**Results:**

A total of 1861 patients were analyzed. In a multivariable logistic regression model, higher propofol doses (Q3, 100.7–153.6 mg/kg) were associated with good functional outcome (OR 1.62, 95%CI 1.12—2.34) and (Q2 and Q3, 43.9–153.6 mg/kg) with survival (OR 1.49, 95%CI 1.05—2.12 and OR 1.84, 95%CI 1.27—2.65, respectively). Receiving fentanyl and remifentanil were associated with good functional outcome (OR 1.69, 95%CI 1.27—2.26 and OR 1.50, 95%CI 1.11—2.02) and survival (OR 1.80, 95%CI 1.35—2.40 and OR 1.56, 95%CI 1.16—2.10). Receiving fentanyl (OR 0.64, 95%CI 0.48—0.86) and higher propofol doses (Q2-4 (43.9–669.4 mg/kg) were associated with the occurrence of clinical seizures. The highest quartile of propofol dose (153.7–669.4 mg/kg, OR 3.19, 95%CI 1.91—5.42) was associated with late awakening.

**Conclusions:**

In this study, higher doses of propofol and the use of remifentanil and fentanyl were associated with good functional outcome and survival, occurrence of clinical seizures, and late awakening.

**Supplementary Information:**

The online version contains supplementary material available at 10.1186/s13054-025-05461-0.

## Introduction

The majority of patients unconscious after out-of-hospital cardiac arrest (OHCA) receive intensive care treatment and targeted temperature management (TTM) [1, 2]. TTM and fever management are recommended to mitigate hypoxic-ischemic brain injury and improve functional outcomes, with sedation and analgesia provided during post-cardiac arrest care to reduce discomfort induced by lowering the body temperature, facilitate therapy, reduce shivering, and prevent awareness during neuromuscular blockade [3–5]. About one third of cardiac arrest patients develop seizures, which are associated with poor outcomes, however, causal relationship with the degree of hypoxic-ischemic brain injury has not been established [6, 7]. Sedatives, some of which are also potent antiepileptic agents, may mitigate secondary brain injury and optimize neurological recovery by lowering metabolic rate and intracranial pressure [8, 9]. Thus, in clinical practice, sedation is often increased when a patient is having clinical seizures or shows signs of pain and agitation.

Sedation carries inherent risks, including compromised circulatory and respiratory functions, increased risk of delirium, prolonged mechanical ventilation, and intensive care stay [10–13]. Importantly, for patients with cardiac arrest, sedation may confound neurological prognostication, influencing the decision on withdrawal of life-sustaining therapies [14, 15].

This study investigates the association between the use of sedatives and analgesics and outcomes, including functional, mortality, clinical seizure, and time to awakening in a large multicenter trial. We hypothesized that there is an association between higher doses of sedatives and analgesics and long-term good functional outcome, survival, occurrence of clinical seizures, and late awakening.

## Methods

This study adheres to the STROBE (Strengthening the Reporting of Observational Studies in Epidemiology) guidelines (Supplement Table 1) [16].

**Table 1:** Background and cardiac arrest characteristics

	**Poor functional outcome**	**Good functional outcome**	**Overall**
n	988	841	1861
Male (%)	737 (74.6)	710 (84.4)	1477 (79.4)
Age (mean (SD))	67.90 (12.01)	59.22 (13.76)	63.8 (13.6)
Frailty score (median (IQR))	3.24 (1.52)	2.14 (0.91)	2 (1)
Body mass index (mean (SD))	27.80 (6.34)	27.12 (4.74)	27.5 (5.7)
Circulatory shock on admission^1^ (%)	359 (36.3)	171 (20.3)	536 (28.8)
Minutes to ROSC (mean (SD))	35.77 (21.21)	24.80 (17.05)	31 (20)
Initial shockable rhythm (%)	585 (59.2)	761 (90.5)	1371 (73.7)
Bystander CPR (%)	737 (74.6)	725 (86.2)	1487 (79.9)
Previous liver disease (%)	31 (3.1)	11(1.3)	43 (2.3)
Previous renal disease (%)	72 (7.3)	19 (2.3)	92 (4.9)
Previous cerebrovascular disease (%)	90 (9.1)	30 (3.6)	120 (6.4)
Admission FOUR motor (%)			
0 No response to pain	830 (91.6)	607 (79.8)	1463 (86.3)
1 Extension response to pain	19 (2.1)	35 (4.6)	54 (3.2)
2 Flexion response to pain	44 (4.9)	68 (8.9)	113 (6.7)
3 Localizes pain	13 (1.4)	48 (6.3)	61 (3.6)
4 Awake and obeying commands	0 (0.0)	3 (0.4)	5 (0.3)

^1^ Circulatory shock at admission was defined as a systolic blood pressure of < 90 mmHg for > 30 min or end-organ hypoperfusion (cool arms and legs, urine output < 30 ml/hour, and heart rate < 60 beats/minute)

Abbreviations: *SD* standard deviation, *BMI* Body mass index, *CPR* Cardiopulmonary resuscitation, *FOUR* Full Outline of Unresponsiveness, *ROSC* Return of spontaneous circulation

### Setting and participants

The TTM2-trial (Clinicaltrials.gov NCT02908308) was an international, multicenter, parallel group, investigator-initiated trial randomizing 1900 adult patients between November 2017 and January 2020, with an OHCA of presumed cardiac cause to targeted hypothermia (33°C) (hypothermia) or normothermia with early treatment of fever (< 37.8°C) (normothermia) [17]. As previously reported, unconscious adult (> 18 years) patients with a presumed cardiac cause and ROSC were eligible for inclusion. The main exclusion criteria were time from ROSC to screening of more than 180 min, unwitnessed cardiac arrest with asystole as the initial rhythm and limitations in care [17].

### Patient management

Patients assigned to hypothermia were cooled to 33°C, and this temperature was maintained for 28 h, followed by rewarming to 37°C for 12 h (intervention period). In the normothermia group, the aim was to maintain a temperature of 37.5°C or less. After the intervention period, normothermia was maintained until 72 h after randomization in all patients who remained unconscious, and sedation was discontinued or tapered according to clinical state. Extubation and wake-up test was attempted at the earliest time possible after the intervention period (40 h), based on standard protocols for discontinuation of mechanical ventilation [18].

Multimodal neurological prognostication was performed by a physician blinded to the intervention no earlier than 96 h after randomization, with strict criteria according to the TTM2-trial study protocol [18]. Decision of withdrawal of life-sustaining therapy (WLST) was at the discretion of the treating physician [18].

Neuron-specific enolase (NSE) sampling was optional and conducted at sites with relevant expertise. The highest NSE value was recorded. Bilirubin and glomerular filtration rate (GFR) were measured daily during the first week of intensive care.

### Sedative and analgesic drugs

During the intervention period of 40 h, deep sedation, defined as Richmond Agitation and Sedation Scale (RASS) -4 to -5, was mandatory in both groups and provided similar treatment in both groups [19] (Supplement Table 2). The trial protocol recommended short-acting drugs, but to comply with the pragmatic nature of the trial was left to the treating physician's discretion or local protocol. Type of drug and cumulative dosing of each sedative and analgesic drug administered up to 72 h after randomization were recorded as follows: midazolam (mg), propofol (mg), dexmedetomidine (mcg), clonidine (mcg), esketamine (mg), ketamine (mg), fentanyl (mcg), morphine (mg), remifentanil (mcg) and oxycodone (mg). In patients in whom neurological prognostication was performed, time of discontinuation of sedatives was recorded.

**Table 2:** Clinical variables and outcomes

	**Poor functional outcome**	**good functional outcome**	**Overall**
n	988	841	1861
Normothermia (%)	493 (49.9)	418 (49.7)	931 (50.0)
Early discontinuation of TTM (%)^1^	241 (25.2)	55 (6.7)	300 (16.6)
Shivering^2^ (%)	270 (28.3)	347 (42.1)	632 (34.9)
Clinical seizures (%)^3^	388 (39.8)	74 (8.8)	464 (25.1)
Highest NSE^3^ (mean (SD))	146.29 (203.59)	30.12 (62.95)	88.5 (161.5)
Lowest GFR (mean (SD))	49.45 (25.89)	66.81 (24.56)	57.7 (26.8)
Highest bilirubin (mean (SD))	20.65 (31.11)	20.05 (23.08)	20.4 (27.6)
Time to extubation, days (median (IQR))	5.64 (7.58)	4.79 (6.54)	3.5 (1.9, 5.9)
Time to wake-up, days^4^ (median (IQR))	5.66 (5.42)	3.55 (3.62)	2.5 (1.8, 4.4)
Neurological prognostication performed according to protocol (%)	472 (47.9)	399 (47.4)	883 (47.5)
Poor prognosis likely at time of neurological prognostication (%)	256 (53.8)	8 (2.0)	264 (29.5)
Time of sedation discontinued before prognostication, hours (median (IQR))	27.20 (42.86)	35.21 (38.88)	14.0 (1.0, 48.0)
ICU length of stay, days (median (IQR))	6.38 (7.96)	7.35 (6.85)	4.9 (2.9, 8.1)
Good neurological outcome (mRS 4–6), n (%)	0 (0.0)	841 (100.0)	873 (47.0)
Survival at six months, n (%)	77 (7.8)	841 (100.0)	950 (50.8)

^1^ Discontinuation of TTM any time during the intervention period of 40 h

^2^ The occurrence of shivering during up to 72 h

^3^ Highest NSE measurement during the ICU stay. Measured in 1018 out of 1861 patients

^4^ Data available in 979 patients out of 1861

Abbreviations: *TTM* Target temperature management, *NSE* Neuron specific enolase, *GFR* Glomerular filtration rate, *ICU* intensive care unit, *SD* standard deviation

Shivering was assessed daily during the intensive care stay using the bedside shivering assessment scale (BSAS) [20] (Supplement Table 3). Shivering was defined as BSAS > 1 anytime up to 72 h after randomization. Prophylactic acetaminophen/paracetamol was recommended. In response to a BSAS > 1, the first measure taken was to increase sedation, and secondarily to administer neuromuscular blocking agent at the discretion of the treating physician The goal was to maintain BSAS 0–1.

**Table 3:** Total doses of sedatives, analgesics, and neuromuscular blockade up to 72 h

	**Good**	**Poor**	*p*-value
	841	988	
Propofol, mg/kg (mean (SD))	115.0 (72.2)	98.0 (79.3)	< 0.001
Propofol, n (%)	767 (91%)	815 (82%)	< 0.001
Midazolam, mg/kg (mean (SD))	2.9 (3.4)	2.7 (6.2)	0.638
Midazolam, n (%)	324 (38%)	372 (38%)	0.737
Midazolam and propofol, n (%)	272 (32%)	271 (27%)	0.025
Midazolam only, n (%)	52 (6%)	101 (10%)	0.002
Remifentanil, mcg/kg (mean (SD))	1.2 (3.92)	0.7 (2.33)	0.042
Remifentanil, n (%)	297 (35%)	335 (34%)	0.561
Fentanyl, mcg/kg (mean (SD))	0.4 (3.94)	0.3 (2.91)	0.544
Fentanyl, n (%)	494 (59%)	456 (46%)	< 0.001
Dexmedetomidine, mcg/kg (mean (SD))	0.02 (0.04)	0.02 (0.02)	0.644
Dexmedetomidine, n (%)	113 (14%)	26 (3%)	< 0.001
Oxycodone, mg/kg (mean (SD))	0.3 (0.37)	0.3 (0.41)	0.972
Oxycodone, n (%)	77 (9%)	35 (4%)	< 0.001
Morphine, mg/kg (mean (SD))	0.8 (1.5)	1.2 (2.3)	0.224
Morphine, n (%)	101 (12%)	120 (12%)	0.986
Any neuromuscular blockade, n (%)	483 (57%)	529 (53%)	0.105

### Primary outcome

A good functional outcome was defined as a modified Rankin Scale (mRS) 0–3 at six months. The mRS ranges from 0 to 6, with 0 representing no symptoms, 1 no clinically significant disability, 2 slight disability, 3 moderate disability, 4 moderately severe disability, 5 severe disability, and 6 death [21].

### Secondary outcomes

#### Survival

Survival status was recorded by six months follow-up.

#### Clinical seizures

Clinical seizures were defined as myoclonus or tonic–clonic seizures, which were assessed daily and recorded as present or absent during the intensive care stay up to seven days after randomization. Seizures were managed according to local protocols, at the discretion of the treating physician.

#### Time to awakening

Awakening was defined as obeying commands, i.e. Full Outline of Unresponsiveness (FOUR) (Supplement Table 4) score motor component of four [22]. FOUR score was recorded daily during the first seven days of intensive care. Time to awakening was recorded, with late awakening defined as occurring after 96 h.

**Table 4:** Multivariable logistic regression and chi-square analysis of clinical factors, sedation, and analgesics on functional outcome, survival, clinical seizures, and late awakening Association of clinical factors, TTM, and total dose of propofol up to 72 h with good functional outcome (mRS 0-3) at six months follow up, survival at six months, clinical seizures, and late awakening in multivariate regression model.

	Good functional outcome			Survival		
Number of complete cases	1477			1495		
Reference variable	Poor functional outcome			Mortality		
Variable	OR	CI	p	OR	CI	p
Normothermia	0.84	0.65—1.08	0.176	0.98	0.76—1.26	0.872
Propofol dose (mg/kg)*			0.01			**0.001**
Propofol quartile 1 (0.01 – 43.8 mg/kg)	–	–	–	–	–	**–**
Propofol quartile 2 (43.9–100.6 mg/kg)	1.38	0.96 – 2.00	0.08	1.49	1.05—2.12	**0.025**
Propofol quartile 3 (100.7–153.6 mg/kg)	1.62	1.12—2.34	**0.01**	1.84	1.27—2.65	**0.001**
Propofol quartile 4 (153.7–669.4 mg/kg)	1.04	0.71—1.51	0.825	1.28	0.88—1.85	0.192
Midazolam	1.01	0.77—1.33	0.943	1.10	0.84—1.45	0.49
Fentanyl	1.69	1.27—2.26	** < 0.001**	1.80	1.35—2.40	** < 0.001**
Remifentanil	1.50	1.11—2.02	**0.008**	1.56	1.16—2.10	**0.003**

	Clinical seizures			Late awakening		
Number of complete cases	1505			826		
Reference variable	No clinical seizures			Early awakening		
Normothermia	0.75	0.58–0.96	**0.022**	0.57	0.40–0.81	**0.002**
Propofol dose (mg/kg)*			** < 0.001**			< 0.001
Propofol quartile 1 (0.01 – 43.8 mg/kg)	–	–	–	–	–	**–**
Propofol quartile 2 (43.9–100.6 mg/kg)	1.53	1.06–2.2	**0.022**	0.78	0.46—1.34	0.369
Propofol quartile 3 (100.7–153.6 mg/kg)	1.56	1.06–2.29	**0.023**	1.33	0.80—2.23	0.279
Propofol quartile 4 (153.7–669.4 mg/kg)	2.82	1.94–4.11	** < 0.001**	3.19	1.91–5.42	** < 0.001**
Midazolam	1.99	1.52–2.61	** < 0.001**	1.98	1.38–2.86	** < 0.001**
Fentanyl	0.64	0.48–0.86	**0.003**	1.26	0.86–1.86	0.246
Remifentanil	1.19	0.89–1.59	0.245	1.20	0.82–1.77	0.352

Bolded values indicate significant associations (p < 0.05)

The multivariable regression model is including variables: age, male sex, time to return of spontaneous circulation, witnessed arrest, shock on admission, shockable rhythm, normothermia, shivering or neuromuscular blockade, lowest glomerular filtration rate, and highest bilirubin. Abbreviations: *OR* Odds ratio, *conf* Confidence interval, *p* *p*-value

*Analysed using Analysis of Variance (ANOVA)

#### Statistical analyses

Patient and cardiac arrest characteristics were used to describe the study cohort. Clinical variables such as temperature management strategy, sedation and analgesia use, intensive care interventions, and outcomes, including a figure describing numbers of patients awake, dead, and comatose from day 1 to 7 post randomization, were also presented to describe the post–cardiac arrest care. Cumulative total doses of sedatives and analgesics were adjusted for body weight, expressed as mg or mcg per kg as appropriate. Continuous variables are presented as median and interquartile range (IQR) or mean and standard deviation (SD). Categorical variables are reported using numbers and percentages. Propofol doses were divided into quartiles (Q1-Q4) to explore dose–response relationships, given observed non-linearity, while administration of midazolam, fentanyl, and remifentanil were evaluated as binary variables (yes/no).

#### Associations between sedative and analgesic use and outcomes: Justification for variables in the multivariable model

We evaluated propofol doses using chi-square statistics to test the associations with outcomes (good functional outcome (mRS 0–3) at 6 months, survival at 6 months, clinical seizures, and late awakening). The association of propofol quartiles, midazolam, fentanyl, remifentanil, and outcomes were also evaluated in an univariable and multivariable logistic regression model with odds ratio (OR with 95% confidence intervals (CI)). The overall test of propofol chi-square test and the multiple logistic regression model, including propofol dose, midazolam, fentanyl, and remifentanil, were adjusted for baseline severity of illness, clinically important variables, and design variables of the TTM2-trial to account for severity of illness and clinical factors potentially influencing the choice and dosage of sedative and analgesics. These included: age, sex, witnessed arrest, shockable rhythm, minutes to ROSC, shock on admission, body mass index (BMI), TTM allocation, shivering or administration of any neuromuscular blockade agent (recorded as yes/no), lowest GFR, and highest bilirubin.

#### Sedation/analgesia by temperature group

Sedative and analgesic doses and proportion of patients in the hypothermia versus the normothermia group were analyzed using Wilcoxon rank sum test or Wilcoxon rank rum exact test and using Pearson’s chi-square test.

#### Sensitivity analysis

Patients comatose at 96 h without clinical seizures were included in the sensitivity analysis, as they would be the population most likely to benefit from optimized sedation strategies. Patients with clinical seizures during intensive care stay were excluded, as their convulsions could significantly impact sedation management and confound the analysis. For the patients who underwent neurological prognostication, the time of sedation discontinuation was recorded, allowing for the calculation of the duration of sedation and the average dose of sedatives and analgesics as dose per kilogram per hour.

## Results

**Fig. 1:**
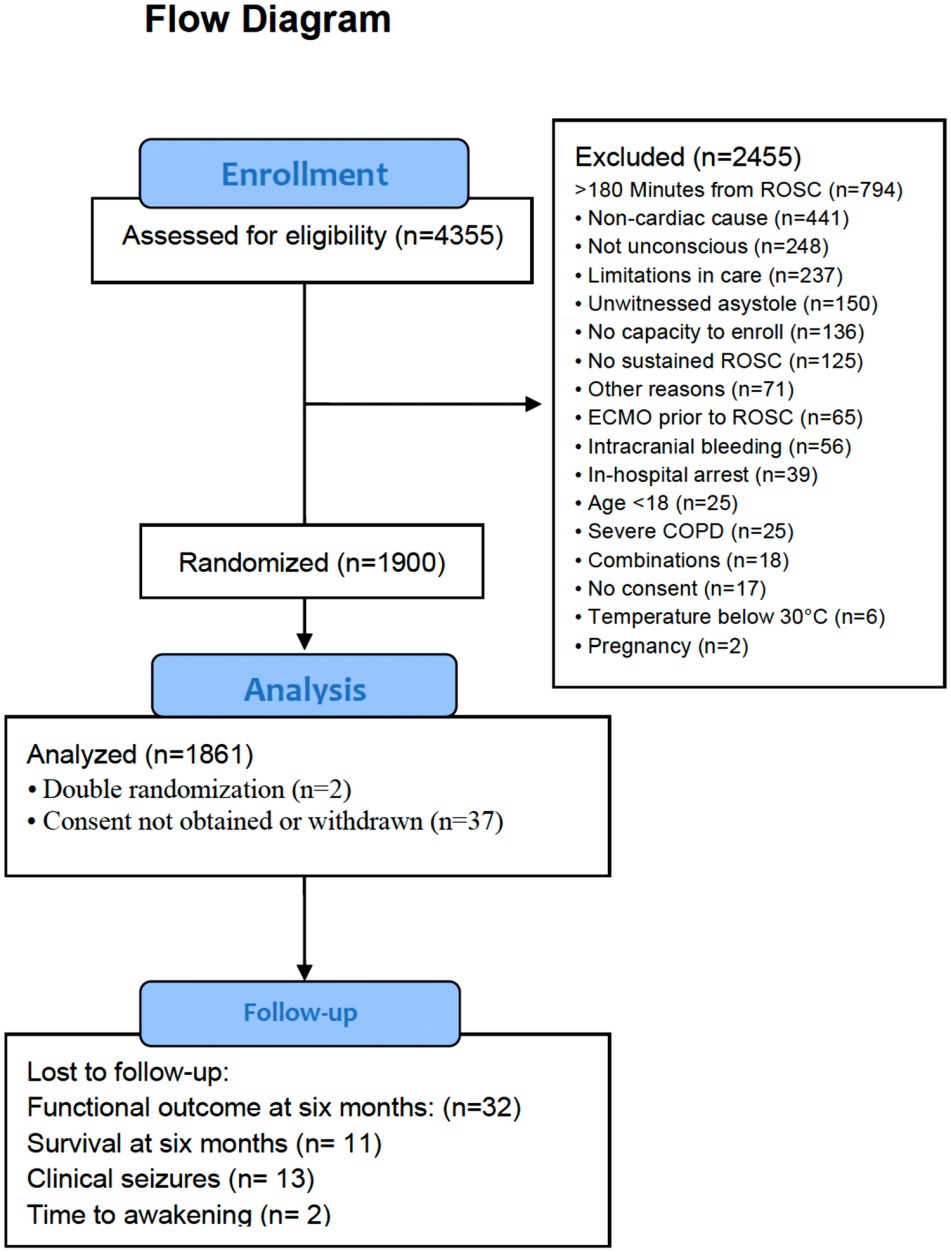
Flow chart of included and excluded patients in this post-hoc study of the TTM2-trial [18]

The TTM2 trial enrolled 1900 patients, with 37 patients withdrawing or unable to provide consent, and 2 patients undergoing randomization twice. Flowchart of included and excluded patients are shown in Fig. 1. This resulted in 1861 patients being included in the intention-to-treat analyses. Detailed patient and cardiac arrest characteristics are summarized in Table 1.

Clinical variables and outcomes are presented in Table 2, while Supplement Fig. 1 describes the proportion of patients awake, comatose, and dead during the first seven days after cardiac arrest. The rate of good functional outcomes (mRS 0–3) at six months was 47.0%, while the six-month survival rate was 50.8%. Clinical seizures were observed in 25% of patients during the intensive care stay. The median time until awakening was 2.5 (IQR 1.8- 4.4) days.

Total sedative and analgesic doses up to 72 h post-randomization are outlined in Table 3. Propofol was the most used sedative, with higher mean doses (115.0 mg/kg vs. 98.0 mg/kg) and proportions of use (91% vs. 82%) in patients with good versus poor outcome, followed by midazolam, which showed similar usage between the two groups (mean dosages 2.9 mg/kg vs. 2.7 mg/kg and both 38%, p = 0.737). Among analgesics, fentanyl was the most frequently, with more frequent use in good outcome patients (mean dosages 0.4 mcg/kg vs 0.3 mcg/kg, proportion of use 60% vs. 47%), followed by remifentanil, which also had a higher mean dosage in good outcome patients (mean dosages 1.2 mcg/kg vs. 0.7 mcg/kg, proportion of use 35% vs. 34%).

There were no significant differences in average doses or proportion of patients receiving any sedative or analgesics between the hypothermia and the normothermia group, as shown in Supplement Table 5. However, significantly more patients received a neuromuscular blockade in the hypothermia group, 614 (66.0%) compared to 418 (44.9%) (p < 0.001).

### Associations between sedatives and outcomes

**Fig. 2:**
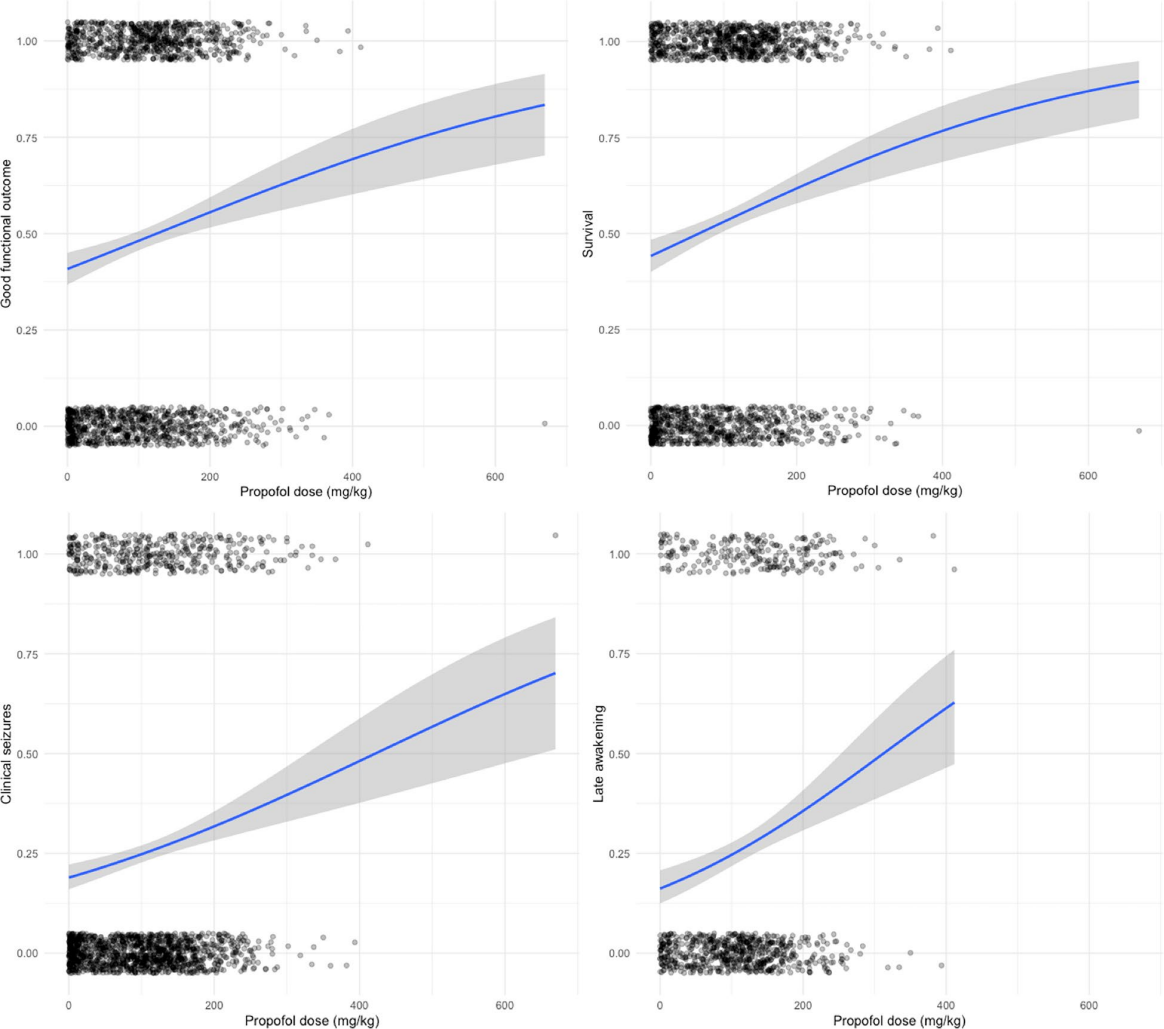
The relationship between cumulative propofol dose and good functional outcome, survival, clinical seizures, and late awakening. A logistic regression was applied to visualize the relationship between cumulative propofol dose (mg/kg) and the probability of good functional outcome, survival, clinical seizures, and late awakening. Each point represents an individual patient (jittered for visibility), with the blue line indicating the predicted probability and the shaded area representing the 95% confidence interval. The increasing curve suggests a non-linear association, with higher cumulative doses associated with a greater probability of the outcome across the observed range

Exploratory analyses indicated a non-linear relationship between propofol dose and outcomes (good functional outcome, survival, clinical seizures, and delayed awakening) in logistic regression models, as illustrated in Fig. 2. Consequently, propofol doses were categorized into quartiles for subsequent analyses to better assess associations with outcomes. Proportion of patients alive at 72 h in each propofol quartile group were: 67.7% (Q1), 88.0% (Q2), 97.0% (Q3), and 99.3% (Q4).

Chi-square analyses showed a significant association between propofol dose and good functional outcome (p < 0.01) and survival at six months (p = 0.001), as well as clinical seizures (p < 0.001), and late awakening (p < 0.001), also after adjusting for confounding factors. The results of the multivariable logistic regression models are presented in Table 4. In a multivariable regression model, higher propofol doses (Q3, 100.7–153.6 mg/kg) were associated with good functional outcome (OR 1.62 CI 1.12—2.34), and Q2 and Q3 (43.9–153.6 mg/kg) with survival (OR 1.49 95%CI 1.05—2.12 and OR 1.84 95%CI 1.27—2.65, respectively). Fentanyl and remifentanil were associated with good functional outcome (OR 1.69 95% CI 1.27—2.26 and OR 1.50 95% CI 1.11—2.02) and survival at six months follow-up (OR 1.80 95%CI 1.35—2.40 and OR 1.56 95%CI 1.16—2.10). Additionally, fentanyl (OR 0.64 95%CI 0.48—0.86) and higher propofol doses (Q2-4, 43.9–669.4 mg/kg) were associated with clinical seizures. Higher doses of propofol (Q4, 153.7–669.4 mg/kg) were associated with late awakening (OR 3.19 95%CI 1.91—5.42). Furthermore, midazolam was associated with clinical seizures (OR 1.99, CI 95% 1.52–2.61) and with late awakening (OR 1.98, CI 95% 1.38–2.86).

In the sensitivity analysis including 463 patients unconscious at 96 h and without any clinical seizures, 359 (78%) patients had neurological prognostication performed, 90 (25%) patients had a poor neurological prognosis, and median duration without sedatives before neurological prognostication was 6.0 h (IQR 1.0, 46.5). Background, cardiac arrest characteristics, clinical variables and outcomes are presented in supplement Tables 6 and 7, and doses of sedative and analgesic are outlined in Supplement Table 8. Chi-square analyses showed no significant association of propofol dose and good functional outcome was observed, while high dose propofol were significantly associated with survival (Supplement Table 9). In a multivariable regression model, higher propofol doses (Q3 and Q4, 1.86–38.86 mg/kg) were associated with good functional outcome (OR 3.15 95%CI 1.29—8.06 and OR 2.78 95%CI 1.15—6.99, respectively), and survival (OR 3.43 95%CI 1.43—8.59 and OR 3.27 95%CI 1.38 – 8.00, respectively). Receiving fentanyl and remifentanil were associated with good functional outcome (OR 2.16 95% CI 1.10—4.39 and OR 2.25 95% CI 1.14—4.52, respectively) and survival (OR 2.65 95%CI 1.35—5.40 and OR 2.25 95%CI 1.15—4.49, respectively).

## Discussion

In this post-hoc analysis of a large multicentered trial comparing hypothermia with normothermia in out of hospital cardiac arrest, including 40 h of protocolized deep sedation, we found a significant association between higher total doses of propofol and good functional outcome and survival at 6 months. Notably, proportion of patients alive at 72 h increased across quartiles of total propofol dose, suggesting a dose–response relationship between higher propofol exposure and early survival. Additionally, higher total doses of propofol over the first 72 h after randomization were associated with a higher frequency of clinical seizures, with high total doses also associated with late awakening. In addition, we found that the use of the two analgesics remifentanil and fentanyl was associated with good functional outcome and survival at 6 months follow-up. In a subgroup of patients without clinically observed seizures and remaining comatose at the time of earliest neurological prognostication (96 h), we found that higher hourly doses of propofol were associated with good functional outcome and survival at 6 months follow-up. Our results also indicate that the use of fentanyl and remifentanil is associated with good functional outcomes and survival, further emphasizing that patients with milder injuries tend to need more sedatives and analgesics for therapeutic comfort.

Seizures are often observed after cardiac arrest as a result of neuronal excitation due to brain injury [6]. Seizures are strongly associated with poor outcomes and if left untreated, can potentially exacerbate brain injury by increasing metabolic demand, disruption of cerebral autoregulation, and excitotoxicity [23–27]. Since the causality cannot be known, it is unclear whether a higher dose of propofol is neuroprotective and reduces the risk of seizures or if this only reflects the clinical practice of increasing the sedation when a patient is having clinical seizures or shows signs of pain and agitation. Thus, in patients without seizures, higher doses of propofol were associated with improved outcomes, aligning with the observation that patients with less severe brain injuries often require more sedatives for comfort. Alternatively, this may suggest that sedation itself has neuroprotective effects. However, the direction of this relationship remains uncertain, highlighting the need for further investigation.

Shivering is another common reason for increased sedation in clinical practice, and in the main trial, it was recommended that shivering be treated with increased sedation as first-line therapy. A shivering response may indicate preserved thermoregulation and less severe brain injury and has been previously associated with improved outcomes after cardiac arrest [28–30]. Higher doses of sedatives and analgesics observed in patients with good functional outcomes may be partly explained by the higher incidence of shivering in patients with good functional outcome and survival [31–33].

In concordance with our findings that midazolam was associated with late awakening, a small randomized clinical trial comparing sedation regimens during hypothermia after cardiac arrest found that propofol and remifentanil significantly reduced time to extubation compared to midazolam and fentanyl [34]. Another trial with 460 participants demonstrated that the use of propofol and remifentanil compared to midazolam and fentanyl resulted in significantly earlier awakening and more ventilator-free days, however, with no differences in survival nor neurological outcomes at hospital discharge [35].

There were no statistically significant differences in sedation or analgesia dosing between patients treated with hypothermia or normothermia. This suggests that doses to keep the patients at deep sedation during TTM were similar. However, after adjusting for sedation and analgesia, severity of illness, and clinical factors, we found normothermia to be associated with a reduced risk of late awakening compared to hypothermia. Hypothermia has previously been shown to be associated with late awakening [36]. Hypothermia decreases drug metabolism and elimination and increases the risk of lingering sedation, which consequently may delay awakening [37–40].

Current guidelines favor the use of short-acting sedatives and analgesics during TTM, specifically propofol, remifentanil, and fentanyl, over midazolam and morphine, and recommend stopping sedatives as soon as possible to assess the level of consciousness and to facilitate neurological prognostication [3–5]. However, limited evidence supports these recommendations, reflected in the variability in clinical practice [41–44]. In this study propofol was the most used sedative agent, however, midazolam was also commonly used. Our multivariable regression model showed no association between midazolam use and good functional outcomes or survival. Instead, midazolam use was associated with seizures and delayed awakening, even after adjusting for illness severity and clinical factors. Other studies have similarly reported significant use of long-acting drugs [45–48]. While causality cannot be established in this study, the use of long-acting sedatives may reflect patient-specific challenges, such as hemodynamic instability or clinical seizures, which influence sedative choice. Alternatively, the selection of sedatives may depend on physician preference or institutional practices [48, 49].

### Strengths and limitations

This study provides the to date most comprehensive evaluation of sedation and analgesia management after out-of-hospital cardiac arrest, with robust adjustments for illness severity. By examining individual patient data, the study identifies factors influencing outcome. The large, diverse patient cohort from international centers further strengthens the generalizability of the findings. Nevertheless, as with all observational analyses, particularly post-hoc studies, there is a risk of confounding bias. Although we adjusted for key clinical variables and illness severity in the multivariable analysis, there may still be residual confounding. For instance, while use of neuromuscular blockade was included in the multivariable models to account for their potential impact, the depth and duration of neuromuscular blockade were not recorded, limiting our ability to assess their relationship with sedation depth, neurological status, or outcomes. Additionally, EEG recordings were not uniformly available across participating centers and were therefor not included in this study which may have introduced heterogeneity in seizure detection. Moreover, variation in sedation and analgesia practices across the 61 participating centers may represent a source of residual confounding not fully captured by the current multivariable model. Although the TTM2-trial protocol was standardized across sites, recommending a target for sedation depth (RASS –4 to –5) and the use of short-acting agents, differences in clinical practice may still have influenced drug choice and dosing, and are not fully accounted for in this analysis.

Furthermore, sedation and analgesia management were not primary outcomes of the original TTM2 trial. As such, there is a risk of post-randomization bias, particularly related to differences in clinical management not captured by available data. To mitigate potential information bias, data was collected prospectively using standardized case report forms across all sites. However, with the post-hoc nature of the study, further investigation to establish the causality of the associations found, and findings should be interpreted as hypothesis-generating.

Additionally, while sedation was mandatory up to 40 h, data on sedative and analgesic cumulative doses were collected at 72 h. The reasons for prolonged sedation or titration of sedation were not recorded, such as the adjustment of sedative and analgesic dosages in the context of WLST. To address this, we conducted subgroup analyses, analyzing those comatose at 96 h and with known sedation duration, to improve our understanding of sedation and analgesia management in post-cardiac arrest care.

**Future aspects** The findings in this study and the possible impact of sedation strategies on patient outcomes, will be further investigated in the Sedation after Cardiac Arrest and Resuscitation (SED-CARE) trial (clinicaltrials.gov no NCT05564754, 2022–10-03). The SED-CARE trial is a part of the CARE platform trial and is essential in addressing the knowledge gap in a randomized and prospective manner, allowing for a more robust assessment of the causal effects of sedation strategies on seizures, neurological outcome, and survival.

## Conclusions

In this post-hoc analysis of a large multicenter cardiac arrest trial, we found that higher doses of propofol were significantly associated with good functional outcomes and survival at six months, clinical seizures, and late awakening. Remifentanil and fentanyl were both associated with good functional outcomes and survival, while midazolam was associated with clinical seizures and delayed awakening. These findings partially support our hypothesis, suggesting that the type and dosage of sedatives and analgesics during post-cardiac arrest care may reflect the severity of illness, with higher doses in patients with less severe brain injury and better outcomes. In comparison, higher doses of sedatives in the presence of seizures are not associated with improve outcomes. Clinical trials are needed to establish causality and optimizing sedation strategies.

## Supplementary Information

Additional file 1

## Data Availability

The datasets used and/or analyzed during the current study are available from the corresponding author on reasonable request. Data is provided within the manuscript or supplementary information files.
