# Supplementary material for: Sedation and analgesia in post-cardiac arrest care: a post hoc analysis of the TTM2 trial
Source: Crit Care. 2025 Jun 17;29:247. doi: 10.1186/s13054-025-05461-0 (PMC12175406; doi:10.1186/s13054-025-05461-0)
Supplement: Supplementary file 1 — Additional file 1 [file 13054_2025_5461_MOESM1_ESM.docx]

# **Sedation and analgesia in post-cardiac arrest care: a post hoc analysis of the TTM2 trial**

# Supplement material

Supplement table 1. STROBE Statement—checklist of items that should be included in reports of observational studies

|  | Item No. | Recommendation | Page  No. | Relevant text from manuscript |
| --- | --- | --- | --- | --- |
| Title and abstract | 1 | (*a*) Indicate the study’s design with a commonly used term in the title or the abstract | 1 |  |
|  |  | (*b*) Provide in the abstract an informative and balanced summary of what was done and what was found | 3 |  |
| Introduction | | | |  |
| Background/rationale | 2 | Explain the scientific background and rationale for the investigation being reported | 4 |  |
| Objectives | 3 | State specific objectives, including any prespecified hypotheses | 4 |  |
| Methods | | | |  |
| Study design | 4 | Present key elements of study design early in the paper | 5 |  |
| Setting | 5 | Describe the setting, locations, and relevant dates, including periods of recruitment, exposure, follow-up, and data collection | 4-5 |  |
| Participants | 6 | (*a*) *Cohort study*—Give the eligibility criteria, and the sources and methods of selection of participants. Describe methods of follow-up  *Case-control study*—Give the eligibility criteria, and the sources and methods of case ascertainment and control selection. Give the rationale for the choice of cases and controls  *Cross-sectional study*—Give the eligibility criteria, and the sources and methods of selection of participants | 4-5 |  |
|  |  | (*b*) *Cohort study*—For matched studies, give matching criteria and number of exposed and unexposed  *Case-control study*—For matched studies, give matching criteria and the number of controls per case |  |  |
| Variables | 7 | Clearly define all outcomes, exposures, predictors, potential confounders, and effect modifiers. Give diagnostic criteria, if applicable | 5-7 |  |
| Data sources/ measurement | 8* | For each variable of interest, give sources of data and details of methods of assessment (measurement). Describe comparability of assessment methods if there is more than one group | *5-7* |  |
| Bias | 9 | Describe any efforts to address potential sources of bias | 8 |  |
| Study size | 10 | Explain how the study size was arrived at | 4-5 |  |

Continued on next page

| Quantitative variables | 11 | Explain how quantitative variables were handled in the analyses. If applicable, describe which groupings were chosen and why | 7-8 |  |
| --- | --- | --- | --- | --- |
| Statistical methods | 12 | (*a*) Describe all statistical methods, including those used to control for confounding | 7-8 |  |
|  |  | (*b*) Describe any methods used to examine subgroups and interactions | 8 |  |
|  |  | (*c*) Explain how missing data were addressed | 8 |  |
|  |  | (*d*) *Cohort study*—If applicable, explain how loss to follow-up was addressed  *Case-control study*—If applicable, explain how matching of cases and controls was addressed  *Cross-sectional study*—If applicable, describe analytical methods taking account of sampling strategy | 8 |  |
|  |  | (*e*) Describe any sensitivity analyses |  |  |
| Results | | | | |
| Participants | 13* | (a) Report numbers of individuals at each stage of study—eg numbers potentially eligible, examined for eligibility, confirmed eligible, included in the study, completing follow-up, and analysed | 8 |  |
|  |  | (b) Give reasons for non-participation at each stage | 8 |  |
|  |  | (c) Consider use of a flow diagram | 8 |  |
| Descriptive data | 14* | (a) Give characteristics of study participants (eg demographic, clinical, social) and information on exposures and potential confounders | 8 (table 1 and 2) |  |
|  |  | (b) Indicate number of participants with missing data for each variable of interest | 9 |  |
|  |  | (c) *Cohort study*—Summarise follow-up time (eg, average and total amount) | 7 |  |
| Outcome data | 15* | *Cohort study*—Report numbers of outcome events or summary measures over time | *7* |  |
|  |  | *Case-control study—*Report numbers in each exposure category, or summary measures of exposure |  |  |
|  |  | *Cross-sectional study—*Report numbers of outcome events or summary measures |  |  |
| Main results | 16 | (*a*) Give unadjusted estimates and, if applicable, confounder-adjusted estimates and their precision (eg, 95% confidence interval). Make clear which confounders were adjusted for and why they were included | 8-10 |  |
|  |  | (*b*) Report category boundaries when continuous variables were categorized |  |  |
|  |  | (*c*) If relevant, consider translating estimates of relative risk into absolute risk for a meaningful time period |  |  |

Continued on next page

| Other analyses | 17 | Report other analyses done—eg analyses of subgroups and interactions, and sensitivity analyses | 10 |  |
| --- | --- | --- | --- | --- |
| Discussion | | | | |
| Key results | 18 | Summarise key results with reference to study objectives | 10 |  |
| Limitations | 19 | Discuss limitations of the study, taking into account sources of potential bias or imprecision. Discuss both direction and magnitude of any potential bias | 13 |  |
| Interpretation | 20 | Give a cautious overall interpretation of results considering objectives, limitations, multiplicity of analyses, results from similar studies, and other relevant evidence | 10-13 |  |
| Generalisability | 21 | Discuss the generalisability (external validity) of the study results | 13 |  |
| Other information | |  | | |
| Funding | 22 | Give the source of funding and the role of the funders for the present study and, if applicable, for the original study on which the present article is based |  |  |

*Give information separately for cases and controls in case-control studies and, if applicable, for exposed and unexposed groups in cohort and cross-sectional studies.

Note: An Explanation and Elaboration article discusses each checklist item and gives methodological background and published examples of transparent reporting. The STROBE checklist is best used in conjunction with this article (freely available on the Web sites of PLoS Medicine at http://www.plosmedicine.org/, Annals of Internal Medicine at http://www.annals.org/, and Epidemiology at http://www.epidem.com/). Information on the STROBE Initiative is available at www.strobe-statement.org.

**Supplement figure 1**. Proportions of patients who were awake, comatose, or dead from day 1 to day 7.

**
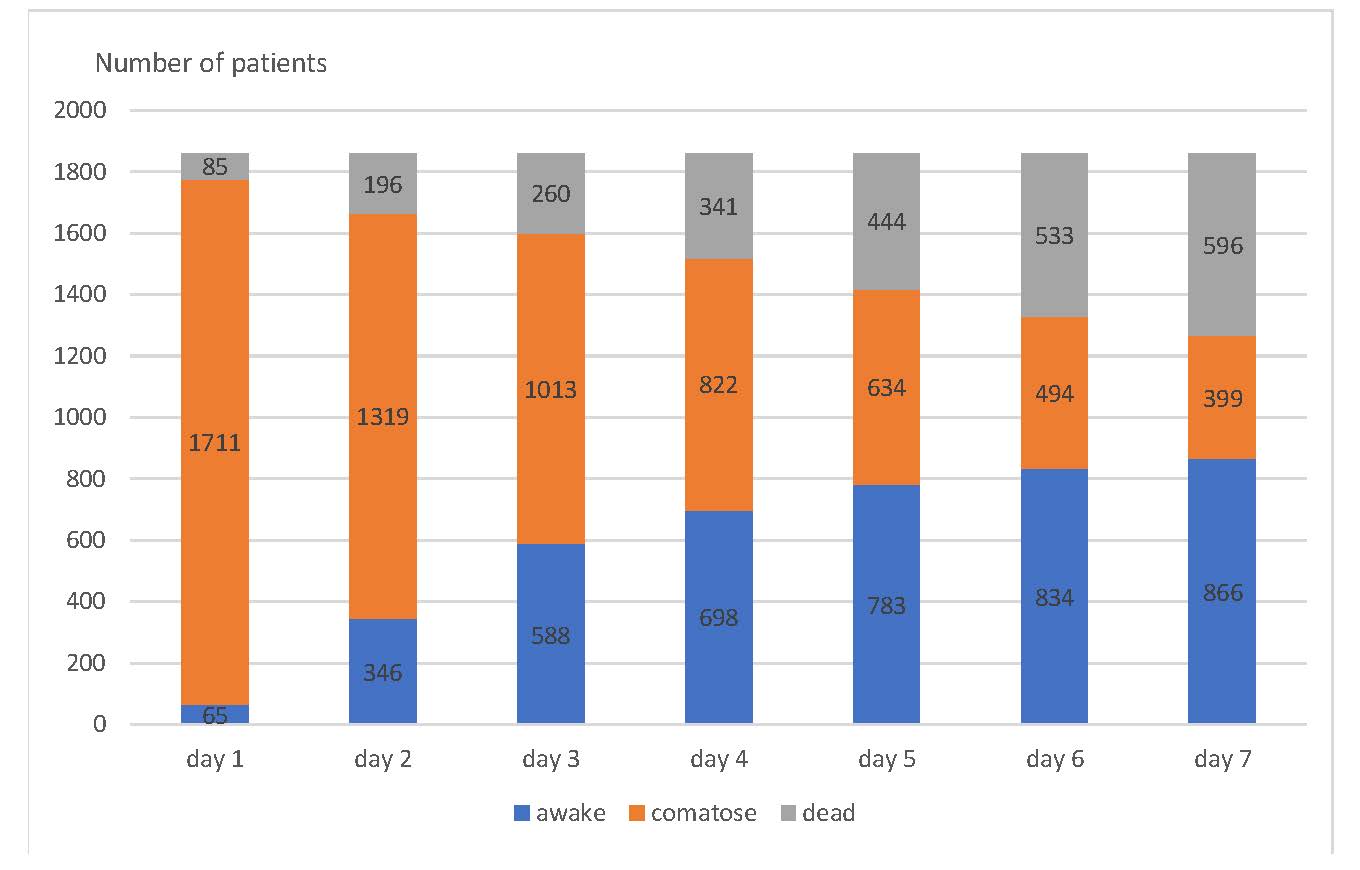
**

Proportions of patients who were awake, comatose, or dead from day 1 to day 7 after cardiac arrest. Data were collected in hour and days from randomization to awakening or death. Patients awake were defined as FOUR motor >4 (obeying commands). Comatose was defined as any patient FOUR motor < 4. The days are defined as every 24 hours period after randomisation: day 1 (0-24 hours), day 2 (24-48 hours), day 3 (48-72 hours), day 4 (72-96 hours), day 5 (96-120 hours), day 6 (120-144 hours), and day 7 (144-168 hours).

## Supplement table 2.

Richmond Agitation and Sedation Scale.

| Score | Classification | Description |
| --- | --- | --- |
| +4 | Combatative | Overtly combative, violent, danger to staff |
| +3 | Very agitated | Pulls or removes tube(s) or catheters; aggressive |
| +2 | Agitated | Frequent non-purposeful movement, fights ventilator |
| +1 | Restless | Anxious, apprehensive, but not aggressive |
| 0 | Alert and calm |  |
| -1 | Drowsy | Awakens to voice (eye opening/contact) > 10 seconds |
| -2 | Light sedation | Briefly awakens to voice (eye opening/contant) < 10 seconds |
| -3 | Moderate sedation | Movement or eye opening. No eye contact |
| -4 | Deep sedation | No response to voice, but movement or eye opening to physical stimulation |
| -5 | Unarousable | No response to voice or physical stimulation |

## Supplement table 3.

Bedside shivering assessment scale.

| Score | Severity | Description |
| --- | --- | --- |
| 0 | None | No shivering |
| 1 | Mild | Shivering localized to neck/thorax, may be seen only as artifact on ECG or felt by palpation |
| 2 | Moderate | Intermittent involvement of the upper extremities ±thorax |
| 3 | Severe | Generalized shivering or sustained upper/lower extremity shivering |

## Supplement table 4.

Full Outline of Responsivness Score Motor response.

| Score | Clinical findings |
| --- | --- |
| 4 | Thumbs-up, fist, or peace sign |
| 3 | Localizing to pain |
| 2 | Flexion response to pain |
| 1 | Extension response to pain |
| 0 | No response to pain or generalized myoclonus status |

Supplement table 5: Doses of sedatives and analgesics
Average doses of sedatives and analgesics up to 72 hours

|  | Hypothermia | Normothermia | p |
| --- | --- | --- | --- |
| n | 930 | 931 |  |
| Propofol (mg/kg) (mean (SD)) | 109.4 (75.1) | 102.8 (77.0) | 0.078 |
| Propofol, n (%) | 792 (85.2) | 819 (88.0) | 0.088 |
| Midazolam (mg/kg) (mean (SD)) | 3.0 (6.5) | 2.5 (3.0) | 0.167 |
| Midazolam, n (%) | 364 (39.1) | 346 (37.2) | 0.407 |
| Midazolam and propofol, n (%) | 273 (29.4) | 282 (30.3) | 0.696 |
| Midazolam only, n (%) | 91 (9.8) | 64 (6.9) | 0.029 |
| Dexmedetomidine (mcg/kg) (mean (SD)) | 0.02 (0.04) | 0.02 (0.03) | 0.876 |
| Dexmedetomidine, n (%) | 66 (7.1) | 78 (8.4) | 0.343 |
| Remifentanil mcg/kg (mean (SD)) | 0.8 (3.0) | 1.1 (3.5) | 0.311 |
| Remifentanil, n (%) | 326 (35.1) | 317 (34.0) | 0.684 |
| Fentanyl (mcg/kg) (mean (SD)) | 0.2 (2.8) | 0.4 (4.0) | 0.431 |
| Fentanyl, n (%) | 495 (53.2) | 477 (51.2) | 0.416 |
| Oxycodone (mg/kg), (mean (SD)) | 0.3 (0.4) | 0.3 (0.3) | 0.484 |
| Oxycodone, n (%) | 50 (5.4) | 63 (6.8) | 0.247 |
| Morphine (mg/kg), (mean (SD)) | 1.1 (1.8) | 0.9 (2.1) | 0.430 |
| Morphine, n (%) | 98 (10.5) | 124 (13.3) | 0.075 |
| Any neuromuscular blockade, n (%) | 614 (66.0) | 418 (44.9) | <0.001 |

Supplement table 6. Background and cardiac arrest characteristics.

|  | **Overall** | **Comatose patients at 96 hours without clinical seizures** |
| --- | --- | --- |
| **n** | 1861 | 463 |
| **Male (%)** | 1477 (79.4) | 378 (81.6) |
| **Age (mean (SD))** | 63.8 (13.6) | 65.5 (12.5) |
| **Frailty score (median (IQR))** | 2 (1) | 2.8 (1.3) |
| **Body mass index (mean (SD))** | 27.5 (5.7) | 27.8 (5.7) |
| **Circulatory shock on admission^1^ (%)** | 536 (28.8) | 143 (30.9) |
| **Minutes to ROSC (mean (SD))** | 31 (20) | 30 (18) |
| **Initial shockable rhythm (%)** | 1371 (73.7) | 351 (75.8) |
| **Bystander CPR (%)** | 1487 (79.9) | 372 (80.3) |
| **Previous liver disease (%)** | 43 (2.3) | 15 (3.2) |
| **Previous renal disease (%)** | 92 (4.9) | 32 (6.9) |
| **Previous cerebrovascular disease (%)** | 120 (6.4) | 29 (6.3) |
| **Admission FOUR motor (%)** |  |  |
| **0 No response to pain** | 1463 (86.3) | 374 (86.6) |
| **1 Extension response to pain** | 54 (3.2) | 11 (2.5) |
| **2 Flexion response to pain** | 113 (6.7) | 30 (6.9) |
| **3 Localizes pain** | 61 (3.6) | 16 (3.7) |
| **4 Awake and obeying commands** | 5 (0.3) | 1 (0.2) |

Supplement table 7. Clinical variables and outcomes.

|  | **Overall** | **Comatose patients at 96 hours without clinical seizures** |
| --- | --- | --- |
| **n** | 1861 | 463 |
| **Normothermia (%)** | 931 (50.0) | 228 (49) |
| **Early discontinuation of TTM (%)** | 300 (16.6) | 21 (4.6) |
| **Shivering^2^ (%)** | 632 (34.9) | 157 (34.7) |
| **Clinical seizures (%)^3^** | 464 (25.1) | 0 (0.0) |
| **Highest NSE^3^ (mean (SD))** | 88.5 (161.5) | 74.4 (114.5) |
| **Lowest GFR (mean (SD))** | 57.7 (26.8) | 54.5 (25.8) |
| **Highest bilirubin (mean (SD))** | 20.4 (27.6) | 21.3 (30.0) |
| **Time to extubation, days (median (IQR))** | 3.5 (1.9, 5.9) | 6.6 (4.7, 11.1) |
| **Time to wake-up, days^4^ (median (IQR))** | 2.5 (1.8, 4.4) | 5.6 (4.4, 8.2) |
| **Neurological prognostication performed according to protocol (%)** | 883 (47.5) | 359 (77.5) |
| **Poor prognosis likely at time of neurological prognostication (%)** | 264 (29.5) | 90 (24.9) |
| **Time of sedation discontinued before prognostication, hours (median (IQR))** | 14.0 (1.0, 48.0) | 6.0 (1.0, 46.5) |
| **ICU length of stay, days (median (IQR))** | 4.9 (2.9, 8.1) | 8.7 (5.8, 13.8) |
| **Good neurological outcome (mRS 4-6), n (%)** | 873 (47.0) | 177 (38.2) |
| **Survival at six months, n (%)** | 950 (50.8) | 262 (56.8) |

## Supplement table 8: Doses of sedatives and analgesics in comatose patients at 96 hours without clinical seizures

Average doses of sedatives and analgesics up to 72 hours in patients with performed neurological prognostication (=with data on duration of sedation and total doses up to 72 hours)

| Functional outcome (modified Rankin Scale) | Poor | Good | P-value |
| --- | --- | --- | --- |
| n | 446 | 375 |  |
| Propofol (mg/kg/h) (mean (SD)) | 2.2 (3.8) | 2.5 (7.5) | 0.509 |
| Propofol, n (%) | 389 (87.2) | 325 (86.7) | 0.896 |
| Midazolam (mg/kg/h) (mean (SD)) | 2.7 (2.8) | 3.9 (3.5) | 0.339 |
| Midazolam, n (%) | 202 (45.3) | 183 (48.8) | 0.351 |
| Midazolam and propofol, n (%) | 156 (35.0) | 147 (39.2) | 0.239 |
| Midazolam only, n (%) | 46 (10.3) | 36 (9.6) | 0.824 |
| Dexmedetomidine (mcg/kg/h) (mean (SD)) | 0.0 (0.0) | 0.0 (0.0) | 0.422 |
| Dexmedetomidine, n (%) | 20 (4.5) | 59 (15.7) | <0.001 |
| Remifentanil mcg/kg/h (mean (SD)) | 0.01 (0.03) | 0.04 (0.15) | 0.034 |
| Remifentanil, n (%) | 172 (38.6) | 132 (35.2) | 0.356 |
| Fentanyl (mcg/kg/h) (mean (SD)) | 0.01 (0.06) | 0.00 (0.00) | 0.345 |
| Fentanyl, n (%) | 215 (48.2) | 214 (57.1) | 0.014 |
| Oxycodone (mg/kg/h), (mean (SD)) | 0.00 (0.01) | 0.01 (0.01) | 0.262 |
| Oxycodone, n (%) | 17 (3.8) | 22 (5.9) | 0.225 |
| Morphine (mg/kg/h), (mean (SD)) | 0.06 (0.22) | 0.02 (0.03) | 0.231 |
| Morphine, n (%) | 49 (11.0) | 34 (9.1) | 0.428 |
| Any neuromuscular blockade, n (%) | 288 (64.6) | 237 (63.2) | 0.737 |

Supplement table 9: Multivariable logistic regression and chi-square analysis of clinical factors, sedation, and analgesics on functional outcome, survival, clinical seizures, and late awakening in comatose patients at 96 hours without seizures.

Chi-square analyses of propofol quartiles and outcomes and multivariable logistic regression model for association of clinical factors, targeted temperature management allocation, and total dose of propofol up to 72 hrs with good functional outcome (mRS 0-3) at six months follow up, survival at six months, clinical seizures, and late awakening in multivariate regression model.

|  | Good functional outcome | | | Survival | | |
| --- | --- | --- | --- | --- | --- | --- |
| Variable | OR | conf | p-value | OR | conf | p-value |
| Propofol dose (mg/kg/h)* |  |  | 0.060 |  |  | 0.020 |
| Propofol quartile 1 (0.01 – 1.02 mg/kg) | - | - | - | - | - | - |
| Propofol quartile 2 (1.03 -1.85 mg/kg/hr) | 1.93 | 0.80 - 4.77 | 0.146 | 1.93 | 0.83 - 4.64 | 0.132 |
| Propofol quartile 3 (1.86-2.55 mg/kg/hr) | 3.15 | 1.29 - 8.06 | 0.014 | 3.43 | 1.43 - 8.59 | 0.007 |
| Propofol quartile 4 (2.56-38.86 mg/kg/hr) | 2.78 | 1.15 - 6.99 | 0.026 | 3.27 | 1.38 - 8 | 0.008 |
| Midazolam | 1.77 | 0.92 - 3.42 | 0.088 | 1.77 | 0.93 - 3.41 | 0.082 |
| Fentanyl | 2.16 | 1.10 - 4.39 | 0.029 | 2.65 | 1.35 - 5.4 | 0.006 |
| Remifentanil | 2.25 | 1.14 - 4.52 | 0.02 | 2.25 | 1.15 - 4.49 | 0.019 |

*The multivariable regression model is including variables: age, male sex, time to return of spontaneous circulation, witnessed arrest, shock on admission, shockable rhythm, normothermia, shivering or neuromuscular blockade, lowest glomerular filtration rate, and highest bilirubin. Abbreviations: OR= Odds ratio, conf= Confidence interval.*

**Chi-square analyses*

## Supplemental data

The missing data for each outcome variable were as follows: neurological outcome, 32 cases (2%); survival, 11 cases (0.6%); clinical seizures, 13 cases (0.7%); and late awakening, 2 cases (1%). Additionally, 35 patients (2%) had missing data for the variable “shivering or neuromuscular blockade.” There were no missing values for the other variables included in the regression models.
